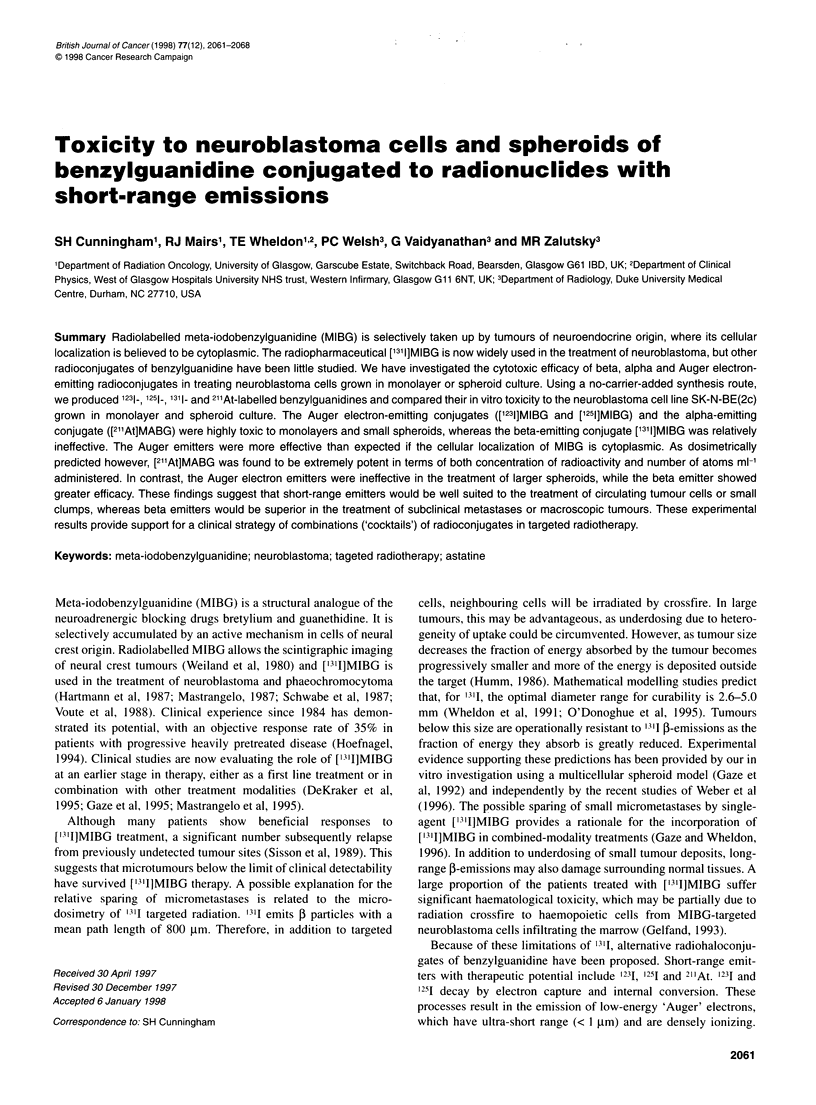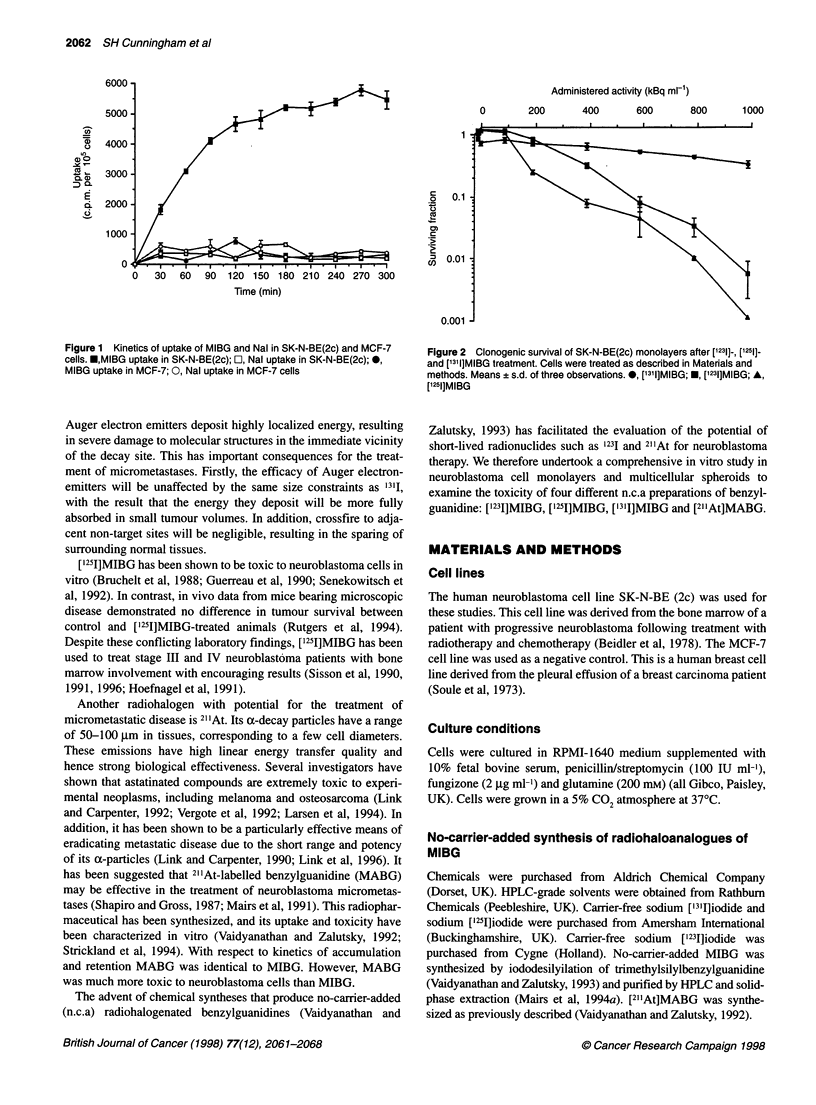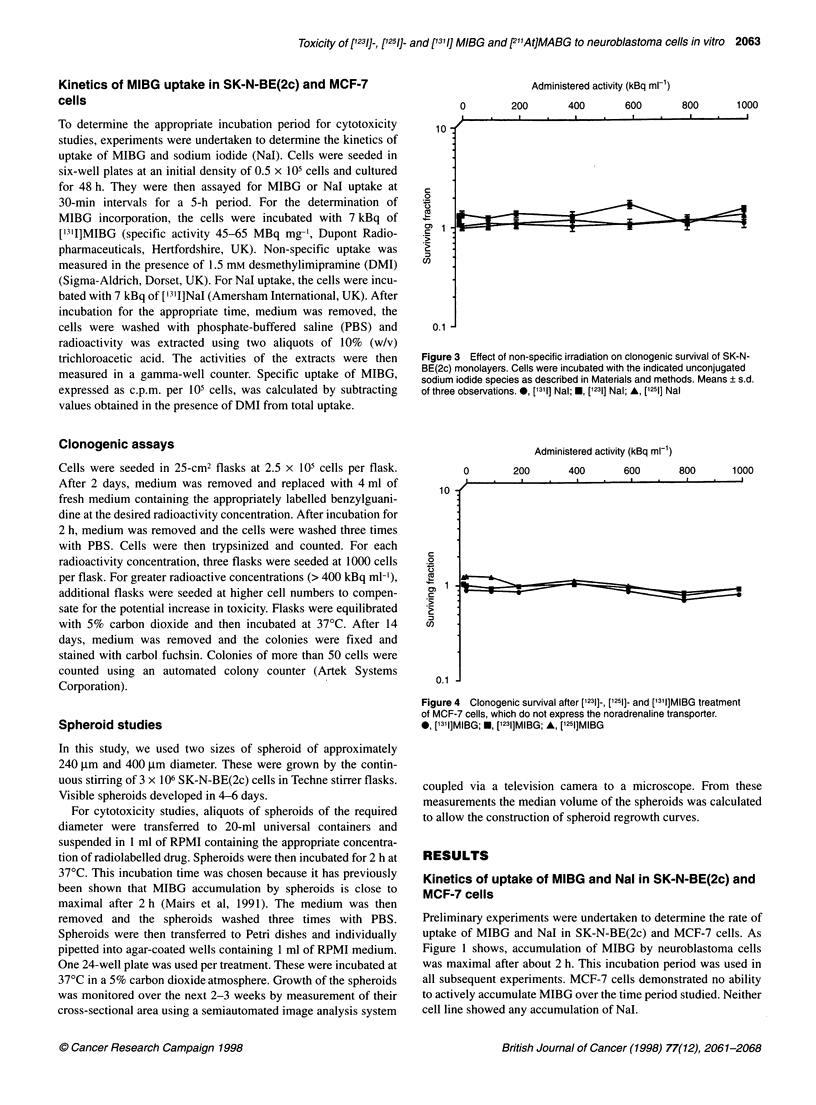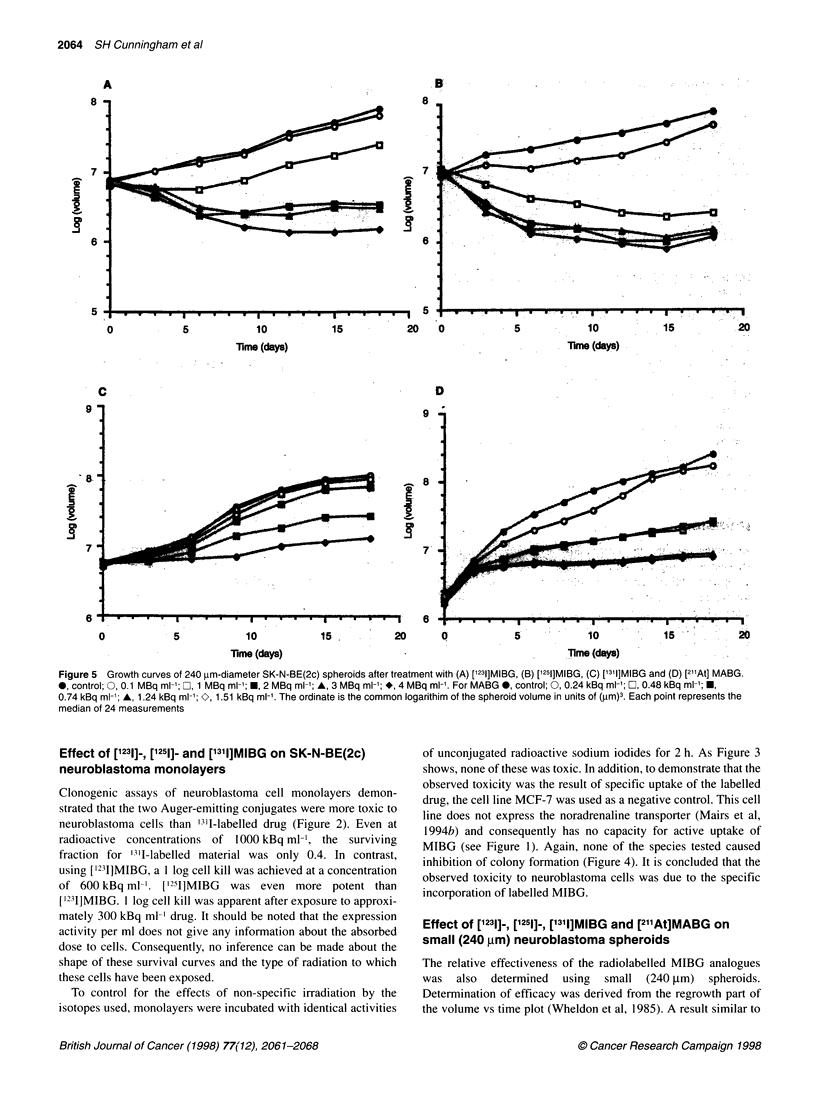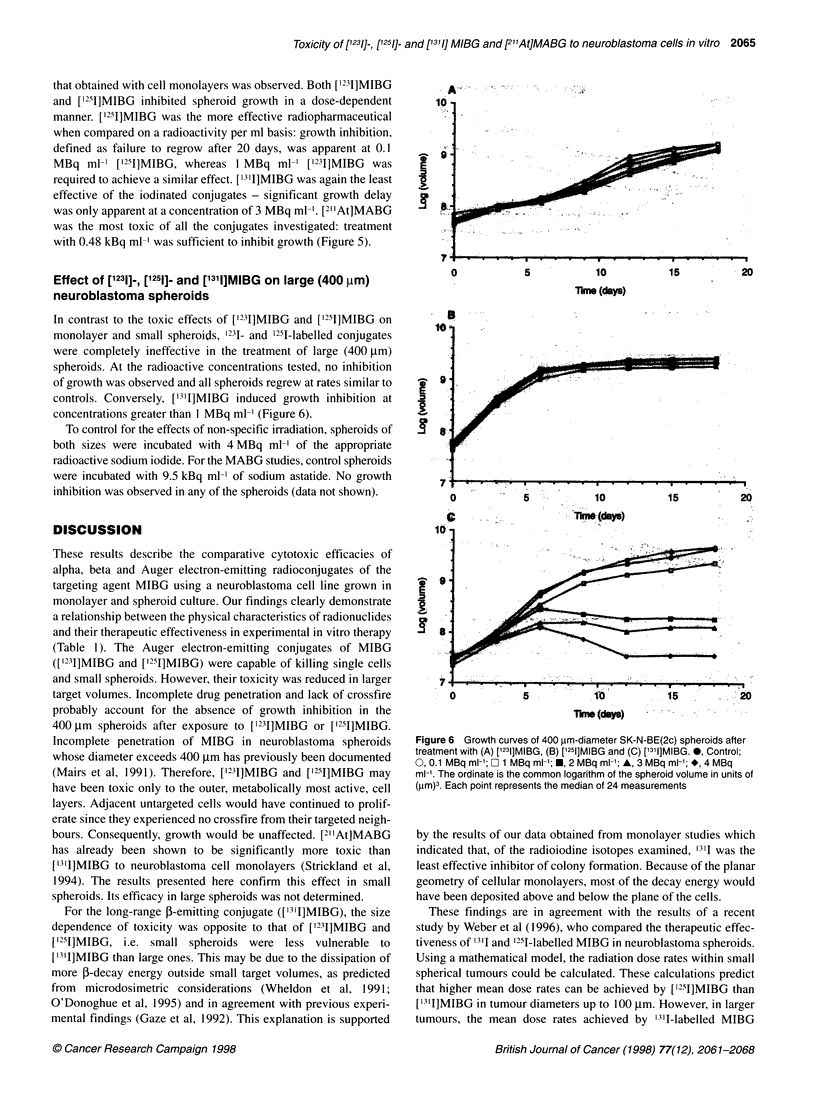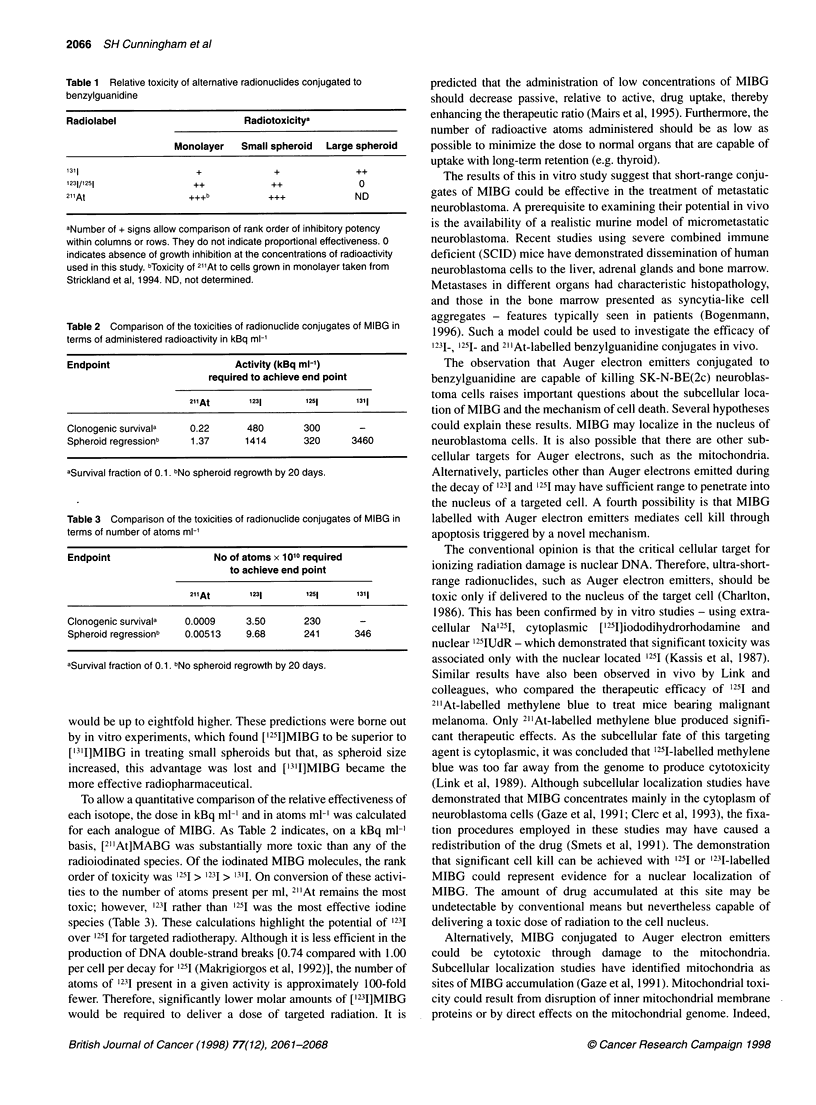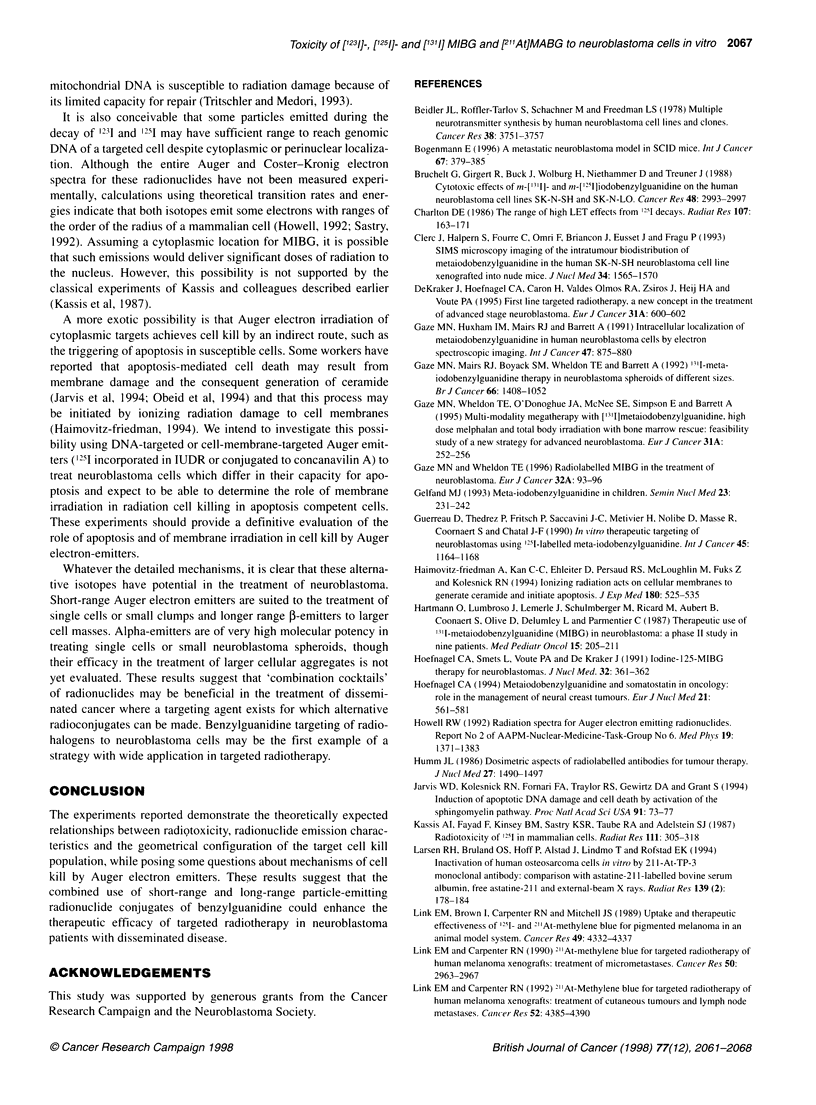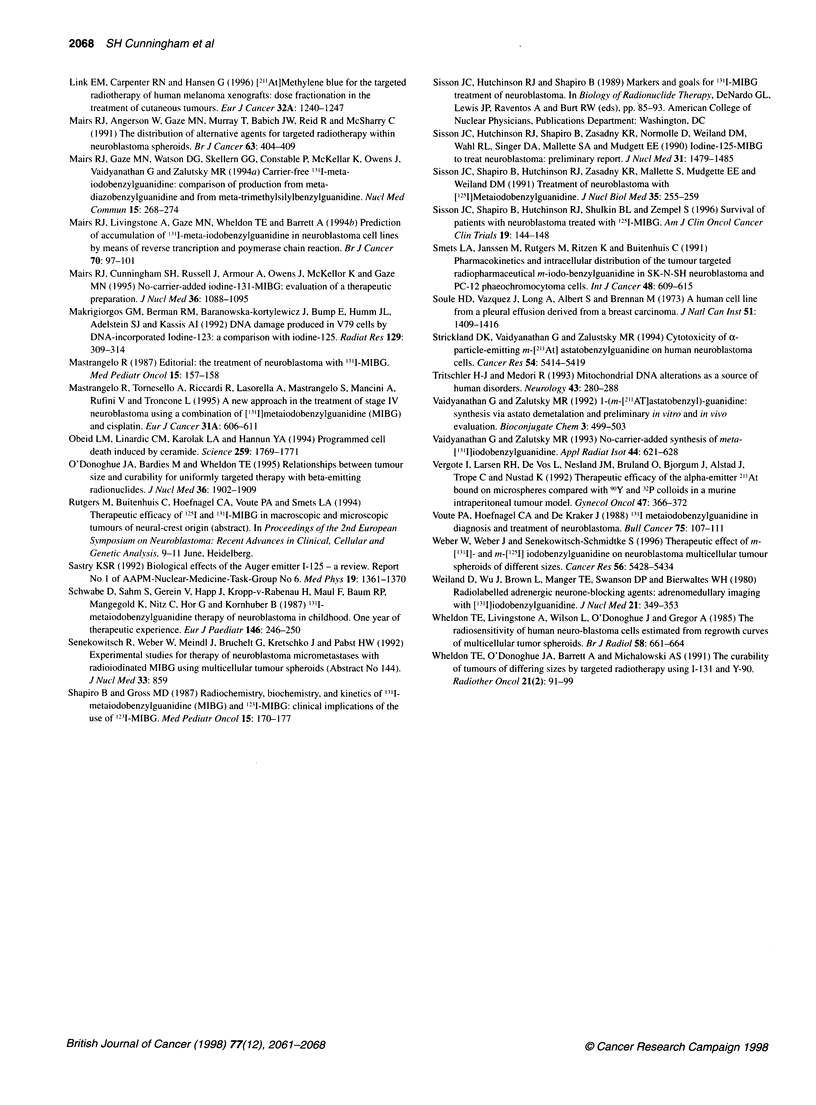# Toxicity to neuroblastoma cells and spheroids of benzylguanidine conjugated to radionuclides with short-range emissions.

**DOI:** 10.1038/bjc.1998.348

**Published:** 1998-06

**Authors:** S. H. Cunningham, R. J. Mairs, T. E. Wheldon, P. C. Welsh, G. Vaidyanathan, M. R. Zalutsky

**Affiliations:** Department of Radiation Oncology, University of Glasgow, UK.

## Abstract

Radiolabelled meta-iodobenzylguanidine (MIBG) is selectively taken up by tumours of neuroendocrine origin, where its cellular localization is believed to be cytoplasmic. The radiopharmaceutical [131I]MIBG is now widely used in the treatment of neuroblastoma, but other radioconjugates of benzylguanidine have been little studied. We have investigated the cytotoxic efficacy of beta, alpha and Auger electron-emitting radioconjugates in treating neuroblastoma cells grown in monolayer or spheroid culture. Using a no-carrier-added synthesis route, we produced 123I-, 125I-, 131I- and 211At-labelled benzylguanidines and compared their in vitro toxicity to the neuroblastoma cell line SK-N-BE(2c) grown in monolayer and spheroid culture. The Auger electron-emitting conjugates ([123I]MIBG and [125I]MIBG) and the alpha-emitting conjugate ([211At]MABG) were highly toxic to monolayers and small spheroids, whereas the beta-emitting conjugate [131I]MIBG was relatively ineffective. The Auger emitters were more effective than expected if the cellular localization of MIBG is cytoplasmic. As dosimetrically predicted however, [211At]MABG was found to be extremely potent in terms of both concentration of radioactivity and number of atoms ml(-1) administered. In contrast, the Auger electron emitters were ineffective in the treatment of larger spheroids, while the beta emitter showed greater efficacy. These findings suggest that short-range emitters would be well suited to the treatment of circulating tumour cells or small clumps, whereas beta emitters would be superior in the treatment of subclinical metastases or macroscopic tumours. These experimental results provide support for a clinical strategy of combinations ('cocktails') of radioconjugates in targeted radiotherapy.


British Journal of Cancer (1998) 77(12), 2061–2068
© 1998 Cancer Research Campaign

### Toxicity to neuroblastoma cells and spheroids of benzylguanidine conjugated to radionuclides with short-range emissions

**SH Cunningham[1], RJ Mairs[1], TE Wheldon[1,2], PC Welsh[3], G Vaidyanathan[3] and MR Zalutsky[3]**

[1]Department of Radiation Oncology, University of Glasgow, Garscube Estate, Switchback Road, Bearsden, Glasgow G61 IBD, UK; [2]Department of Clinical Physics, West of Glasgow Hospitals University NHS trust, Western Infirmary, Glasgow G11 6NT, UK; [3]Department of Radiology, Duke University Medical Centre, Durham, NC 27710, USA

**Summary** Radiolabelled meta-iodobenzylguanidine (MIBG) is selectively taken up by tumours of neuroendocrine origin, where its cellular localization is believed to be cytoplasmic. The radiopharmaceutical [$^{131}$I]MIBG is now widely used in the treatment of neuroblastoma, but other radioconjugates of benzylguanidine have been little studied. We have investigated the cytotoxic efficacy of beta, alpha and Auger electron-emitting radioconjugates in treating neuroblastoma cells grown in monolayer or spheroid culture. Using a no-carrier-added synthesis route, we produced $^{123}$I-, $^{125}$I-, $^{131}$I- and $^{211}$At-labelled benzylguanidines and compared their in vitro toxicity to the neuroblastoma cell line SK-N-BE(2c) grown in monolayer and spheroid culture. The Auger electron-emitting conjugates ([$^{123}$I]MIBG and [$^{125}$I]MIBG) and the alpha-emitting conjugate ([$^{211}$At]MABG) were highly toxic to monolayers and small spheroids, whereas the beta-emitting conjugate [$^{131}$I]MIBG was relatively ineffective. The Auger emitters were more effective than expected if the cellular localization of MIBG is cytoplasmic. As dosimetrically predicted however, [$^{211}$At]MABG was found to be extremely potent in terms of both concentration of radioactivity and number of atoms $ml^{-1}$ administered. In contrast, the Auger electron emitters were ineffective in the treatment of larger spheroids, while the beta emitter showed greater efficacy. These findings suggest that short-range emitters would be well suited to the treatment of circulating tumour cells or small clumps, whereas beta emitters would be superior in the treatment of subclinical metastases or macroscopic tumours. These experimental results provide support for a clinical strategy of combinations ('cocktails') of radioconjugates in targeted radiotherapy.

**Keywords:** meta-iodobenzylguanidine; neuroblastoma; tageted radiotherapy; astatine

Meta-iodobenzylguanidine (MIBG) is a structural analogue of the neuroadrenergic blocking drugs bretylium and guanethidine. It is selectively accumulated by an active mechanism in cells of neural crest origin. Radiolabelled MIBG allows the scintigraphic imaging of neural crest tumours (Weiland et al, 1980) and [$^{131}$I]MIBG is used in the treatment of neuroblastoma and phaeochromocytoma (Hartmann et al, 1987; Mastrangelo, 1987; Schwabe et al, 1987; Voute et al, 1988). Clinical experience since 1984 has demonstrated its potential, with an objective response rate of 35% in patients with progressive heavily pretreated disease (Hoefnagel, 1994). Clinical studies are now evaluating the role of [$^{131}$I]MIBG at an earlier stage in therapy, either as a first line treatment or in combination with other treatment modalities (DeKraker et al, 1995; Gaze et al, 1995; Mastrangelo et al, 1995).

Although many patients show beneficial responses to [$^{131}$I]MIBG treatment, a significant number subsequently relapse from previously undetected tumour sites (Sisson et al, 1989). This suggests that microtumours below the limit of clinical detectability have survived [$^{131}$I]MIBG therapy. A possible explanation for the relative sparing of micrometastases is related to the microdosimetry of $^{131}$I targeted radiation. $^{131}$I emits β particles with a mean path length of 800 μm. Therefore, in addition to targeted cells, neighbouring cells will be irradiated by crossfire. In large tumours, this may be advantageous, as underdosing due to heterogeneity of uptake could be circumvented. However, as tumour size decreases the fraction of energy absorbed by the tumour becomes progressively smaller and more of the energy is deposited outside the target (Humm, 1986). Mathematical modelling studies predict that, for $^{131}$I, the optimal diameter range for curability is 2.6–5.0 mm (Wheldon et al, 1991; O'Donoghue et al, 1995). Tumours below this size are operationally resistant to $^{131}$I β-emissions as the fraction of energy they absorb is greatly reduced. Experimental evidence supporting these predictions has been provided by our in vitro investigation using a multicellular spheroid model (Gaze et al, 1992) and independently by the recent studies of Weber et al (1996). The possible sparing of small micrometastases by single-agent [$^{131}$I]MIBG provides a rationale for the incorporation of [$^{131}$I]MIBG in combined-modality treatments (Gaze and Wheldon, 1996). In addition to underdosing of small tumour deposits, long-range β-emissions may also damage surrounding normal tissues. A large proportion of the patients treated with [$^{131}$I]MIBG suffer significant haematological toxicity, which may be partially due to radiation crossfire to haemopoietic cells from MIBG-targeted neuroblastoma cells infiltrating the marrow (Gelfand, 1993).

Because of these limitations of $^{131}$I, alternative radiohaloconjugates of benzylguanidine have been proposed. Short-range emitters with therapeutic potential include $^{123}$I, $^{125}$I and $^{211}$At. $^{123}$I and $^{125}$I decay by electron capture and internal conversion. These processes result in the emission of low-energy 'Auger' electrons, which have ultra-short range (< 1 μm) and are densely ionizing.

*Received 30 April 1997*
*Revised 30 December 1997*
*Accepted 6 January 1998*

*Correspondence to:* SH Cunningham

Figure 1 Kinetics of uptake of MIBG and NaI in SK-N-BE(2c) and MCF-7 cells. ■,MIBG uptake in SK-N-BE(2c); □, NaI uptake in SK-N-BE(2c); ●, MIBG uptake in MCF-7; ○, NaI uptake in MCF-7 cells

Figure 2 Clonogenic survival of SK-N-BE(2c) monolayers after [$^{123}$I]-, [$^{125}$I]- and [$^{131}$I]MIBG treatment. Cells were treated as described in Materials and methods. Means ± s.d. of three observations. ●, [$^{131}$I]MIBG; ■, [$^{123}$I]MIBG; ▲, [$^{125}$I]MIBG

Auger electron emitters deposit highly localized energy, resulting in severe damage to molecular structures in the immediate vicinity of the decay site. This has important consequences for the treatment of micrometastases. Firstly, the efficacy of Auger electron-emitters will be unaffected by the same size constraints as $^{131}$I, with the result that the energy they deposit will be more fully absorbed in small tumour volumes. In addition, crossfire to adjacent non-target sites will be negligible, resulting in the sparing of surrounding normal tissues.

[$^{125}$I]MIBG has been shown to be toxic to neuroblastoma cells in vitro (Bruchelt et al, 1988; Guerreau et al, 1990; Senekowitsch et al, 1992). In contrast, in vivo data from mice bearing microscopic disease demonstrated no difference in tumour survival between control and [$^{125}$I]MIBG-treated animals (Rutgers et al, 1994). Despite these conflicting laboratory findings, [$^{125}$I]MIBG has been used to treat stage III and IV neuroblastoma patients with bone marrow involvement with encouraging results (Sisson et al, 1990, 1991, 1996; Hoefnagel et al, 1991).

Another radiohalogen with potential for the treatment of micrometastatic disease is $^{211}$At. Its α-decay particles have a range of 50–100 μm in tissues, corresponding to a few cell diameters. These emissions have high linear energy transfer quality and hence strong biological effectiveness. Several investigators have shown that astatinated compounds are extremely toxic to experimental neoplasms, including melanoma and osteosarcoma (Link and Carpenter, 1992; Vergote et al, 1992; Larsen et al, 1994). In addition, it has been shown to be a particularly effective means of eradicating metastatic disease due to the short range and potency of its α-particles (Link and Carpenter, 1990; Link et al, 1996). It has been suggested that $^{211}$At-labelled benzylguanidine (MABG) may be effective in the treatment of neuroblastoma micrometastases (Shapiro and Gross, 1987; Mairs et al, 1991). This radiopharmaceutical has been synthesized, and its uptake and toxicity have been characterized in vitro (Vaidyanathan and Zalutsky, 1992; Strickland et al, 1994). With respect to kinetics of accumulation and retention MABG was identical to MIBG. However, MABG was much more toxic to neuroblastoma cells than MIBG.

The advent of chemical syntheses that produce no-carrier-added (n.c.a) radiohalogenated benzylguanidines (Vaidyanathan and Zalutsky, 1993) has facilitated the evaluation of the potential of short-lived radionuclides such as $^{123}$I and $^{211}$At for neuroblastoma therapy. We therefore undertook a comprehensive in vitro study in neuroblastoma cell monolayers and multicellular spheroids to examine the toxicity of four different n.c.a preparations of benzylguanidine: [$^{123}$I]MIBG, [$^{125}$I]MIBG, [$^{131}$I]MIBG and [$^{211}$At]MABG.

### MATERIALS AND METHODS

### Cell lines

The human neuroblastoma cell line SK-N-BE (2c) was used for these studies. This cell line was derived from the bone marrow of a patient with progressive neuroblastoma following treatment with radiotherapy and chemotherapy (Beidler et al, 1978). The MCF-7 cell line was used as a negative control. This is a human breast cell line derived from the pleural effusion of a breast carcinoma patient (Soule et al, 1973).

### Culture conditions

Cells were cultured in RPMI-1640 medium supplemented with 10% fetal bovine serum, penicillin/streptomycin (100 IU ml$^{-1}$), fungizone (2 μg ml$^{-1}$) and glutamine (200 mM) (all Gibco, Paisley, UK). Cells were grown in a 5% $CO_2$ atmosphere at 37°C.

### No-carrier-added synthesis of radiohaloanalogues of MIBG

Chemicals were purchased from Aldrich Chemical Company (Dorset, UK). HPLC-grade solvents were obtained from Rathburn Chemicals (Peebleshire, UK). Carrier-free sodium [$^{131}$I]iodide and sodium [$^{125}$I]iodide were purchased from Amersham International (Buckinghamshire, UK). Carrier-free sodium [$^{123}$I]iodide was purchased from Cygne (Holland). No-carrier-added MIBG was synthesized by iododesilyilation of trimethylsilylbenzylguanidine (Vaidyanathan and Zalutsky, 1993) and purified by HPLC and solid-phase extraction (Mairs et al, 1994*a*). [$^{211}$At]MABG was synthesized as previously described (Vaidyanathan and Zalutsky, 1992).

© Cancer Research Campaign 1998

### Kinetics of MIBG uptake in SK-N-BE(2c) and MCF-7 cells

To determine the appropriate incubation period for cytotoxicity studies, experiments were undertaken to determine the kinetics of uptake of MIBG and sodium iodide (NaI). Cells were seeded in six-well plates at an initial density of $0.5 \times 10^5$ cells and cultured for 48 h. They were then assayed for MIBG or NaI uptake at 30-min intervals for a 5-h period. For the determination of MIBG incorporation, the cells were incubated with 7 kBq of [$^{131}$I]MIBG (specific activity 45–65 MBq mg$^{-1}$, Dupont Radiopharmaceuticals, Hertfordshire, UK). Non-specific uptake was measured in the presence of 1.5 mM desmethylimipramine (DMI) (Sigma-Aldrich, Dorset, UK). For NaI uptake, the cells were incubated with 7 kBq of [$^{131}$I]NaI (Amersham International, UK). After incubation for the appropriate time, medium was removed, the cells were washed with phosphate-buffered saline (PBS) and radioactivity was extracted using two aliquots of 10% (w/v) trichloroacetic acid. The activities of the extracts were then measured in a gamma-well counter. Specific uptake of MIBG, expressed as c.p.m. per $10^5$ cells, was calculated by subtracting values obtained in the presence of DMI from total uptake.

**Figure 3** Effect of non-specific irradiation on clonogenic survival of SK-N-BE(2c) monolayers. Cells were incubated with the indicated unconjugated sodium iodide species as described in Materials and methods. Means ± s.d. of three observations. ●, [$^{131}$I] NaI; ■, [$^{123}$I] NaI; ▲, [$^{125}$I] NaI

### Clonogenic assays

Cells were seeded in 25-cm$^2$ flasks at $2.5 \times 10^5$ cells per flask. After 2 days, medium was removed and replaced with 4 ml of fresh medium containing the appropriately labelled benzylguanidine at the desired radioactivity concentration. After incubation for 2 h, medium was removed and the cells were washed three times with PBS. Cells were then trypsinized and counted. For each radioactivity concentration, three flasks were seeded at 1000 cells per flask. For greater radioactive concentrations (> 400 kBq ml$^{-1}$), additional flasks were seeded at higher cell numbers to compensate for the potential increase in toxicity. Flasks were equilibrated with 5% carbon dioxide and then incubated at 37°C. After 14 days, medium was removed and the colonies were fixed and stained with carbol fuchsin. Colonies of more than 50 cells were counted using an automated colony counter (Artek Systems Corporation).

**Figure 4** Clonogenic survival after [$^{123}$I]-, [$^{125}$I]- and [$^{131}$I]MIBG treatment of MCF-7 cells, which do not express the noradrenaline transporter. ●, [$^{131}$I]MIBG; ■, [$^{123}$I]MIBG; ▲, [$^{125}$I]MIBG

### Spheroid studies

In this study, we used two sizes of spheroid of approximately 240 µm and 400 µm diameter. These were grown by the continuous stirring of $3 \times 10^6$ SK-N-BE(2c) cells in Techne stirrer flasks. Visible spheroids developed in 4–6 days.

For cytotoxicity studies, aliquots of spheroids of the required diameter were transferred to 20-ml universal containers and suspended in 1 ml of RPMI containing the appropriate concentration of radiolabelled drug. Spheroids were then incubated for 2 h at 37°C. This incubation time was chosen because it has previously been shown that MIBG accumulation by spheroids is close to maximal after 2 h (Mairs et al, 1991). The medium was then removed and the spheroids washed three times with PBS. Spheroids were then transferred to Petri dishes and individually pipetted into agar-coated wells containing 1 ml of RPMI medium. One 24-well plate was used per treatment. These were incubated at 37°C in a 5% carbon dioxide atmosphere. Growth of the spheroids was monitored over the next 2–3 weeks by measurement of their cross-sectional area using a semiautomated image analysis system coupled via a television camera to a microscope. From these measurements the median volume of the spheroids was calculated to allow the construction of spheroid regrowth curves.

### RESULTS

### Kinetics of uptake of MIBG and NaI in SK-N-BE(2c) and MCF-7 cells

Preliminary experiments were undertaken to determine the rate of uptake of MIBG and NaI in SK-N-BE(2c) and MCF-7 cells. As Figure 1 shows, accumulation of MIBG by neuroblastoma cells was maximal after about 2 h. This incubation period was used in all subsequent experiments. MCF-7 cells demonstrated no ability to actively accumulate MIBG over the time period studied. Neither cell line showed any accumulation of NaI.

© Cancer Research Campaign 1998

**Figure 5** Growth curves of 240 μm-diameter SK-N-BE(2c) spheroids after treatment with (A) [$^{123}$I]MIBG, (B) [$^{125}$I]MIBG, (C) [$^{131}$I]MIBG and (D) [$^{211}$At] MABG. ●, control; ○, 0.1 MBq ml$^{-1}$; □, 1 MBq ml$^{-1}$; ■, 2 MBq ml$^{-1}$; ▲, 3 MBq ml$^{-1}$; ◆, 4 MBq ml$^{-1}$. For MABG ●, control; ○, 0.24 kBq ml$^{-1}$; □, 0.48 kBq ml$^{-1}$; ■, 0.74 kBq ml$^{-1}$; ▲, 1.24 kBq ml$^{-1}$; ◇, 1.51 kBq ml$^{-1}$. The ordinate is the common logarithim of the spheroid volume in units of (μm)$^3$. Each point represents the median of 24 measurements

### Effect of [$^{123}$I]-, [$^{125}$I]- and [$^{131}$I]MIBG on SK-N-BE(2c) neuroblastoma monolayers

Clonogenic assays of neuroblastoma cell monolayers demonstrated that the two Auger-emitting conjugates were more toxic to neuroblastoma cells than $^{131}$I-labelled drug (Figure 2). Even at radioactive concentrations of 1000 kBq ml$^{-1}$, the surviving fraction for $^{131}$I-labelled material was only 0.4. In contrast, using [$^{123}$I]MIBG, a 1 log cell kill was achieved at a concentration of 600 kBq ml$^{-1}$. [$^{125}$I]MIBG was even more potent than [$^{123}$I]MIBG. 1 log cell kill was apparent after exposure to approximately 300 kBq ml$^{-1}$ drug. It should be noted that the expression activity per ml does not give any information about the absorbed dose to cells. Consequently, no inference can be made about the shape of these survival curves and the type of radiation to which these cells have been exposed.

To control for the effects of non-specific irradiation by the isotopes used, monolayers were incubated with identical activities of unconjugated radioactive sodium iodides for 2 h. As Figure 3 shows, none of these was toxic. In addition, to demonstrate that the observed toxicity was the result of specific uptake of the labelled drug, the cell line MCF-7 was used as a negative control. This cell line does not express the noradrenaline transporter (Mairs et al, 1994*b*) and consequently has no capacity for active uptake of MIBG (see Figure 1). Again, none of the species tested caused inhibition of colony formation (Figure 4). It is concluded that the observed toxicity to neuroblastoma cells was due to the specific incorporation of labelled MIBG.

### Effect of [$^{123}$I]-, [$^{125}$I]-, [$^{131}$I]MIBG and [$^{211}$At]MABG on small (240 μm) neuroblastoma spheroids

The relative effectiveness of the radiolabelled MIBG analogues was also determined using small (240 μm) spheroids. Determination of efficacy was derived from the regrowth part of the volume vs time plot (Wheldon et al, 1985). A result similar to

*© Cancer Research Campaign 1998*

that obtained with cell monolayers was observed. Both [$^{123}$I]MIBG and [$^{125}$I]MIBG inhibited spheroid growth in a dose-dependent manner. [$^{125}$I]MIBG was the more effective radiopharmaceutical when compared on a radioactivity per ml basis: growth inhibition, defined as failure to regrow after 20 days, was apparent at 0.1 MBq ml$^{-1}$ [$^{125}$I]MIBG, whereas 1 MBq ml$^{-1}$ [$^{123}$I]MIBG was required to achieve a similar effect. [$^{131}$I]MIBG was again the least effective of the iodinated conjugates – significant growth delay was only apparent at a concentration of 3 MBq ml$^{-1}$. [$^{211}$At]MABG was the most toxic of all the conjugates investigated: treatment with 0.48 kBq ml$^{-1}$ was sufficient to inhibit growth (Figure 5).

### Effect of [$^{123}$I]-, [$^{125}$I]- and [$^{131}$I]MIBG on large (400 μm) neuroblastoma spheroids

In contrast to the toxic effects of [$^{123}$I]MIBG and [$^{125}$I]MIBG on monolayer and small spheroids, $^{123}$I- and $^{125}$I-labelled conjugates were completely ineffective in the treatment of large (400 μm) spheroids. At the radioactive concentrations tested, no inhibition of growth was observed and all spheroids regrew at rates similar to controls. Conversely, [$^{131}$I]MIBG induced growth inhibition at concentrations greater than 1 MBq ml$^{-1}$ (Figure 6).

To control for the effects of non-specific irradiation, spheroids of both sizes were incubated with 4 MBq ml$^{-1}$ of the appropriate radioactive sodium iodide. For the MABG studies, control spheroids were incubated with 9.5 kBq ml$^{-1}$ of sodium astatide. No growth inhibition was observed in any of the spheroids (data not shown).

### DISCUSSION

These results describe the comparative cytotoxic efficacies of alpha, beta and Auger electron-emitting radioconjugates of the targeting agent MIBG using a neuroblastoma cell line grown in monolayer and spheroid culture. Our findings clearly demonstrate a relationship between the physical characteristics of radionuclides and their therapeutic effectiveness in experimental in vitro therapy (Table 1). The Auger electron-emitting conjugates of MIBG ([$^{123}$I]MIBG and [$^{125}$I]MIBG) were capable of killing single cells and small spheroids. However, their toxicity was reduced in larger target volumes. Incomplete drug penetration and lack of crossfire probably account for the absence of growth inhibition in the 400 μm spheroids after exposure to [$^{123}$I]MIBG or [$^{125}$I]MIBG. Incomplete penetration of MIBG in neuroblastoma spheroids whose diameter exceeds 400 μm has previously been documented (Mairs et al, 1991). Therefore, [$^{123}$I]MIBG and [$^{125}$I]MIBG may have been toxic only to the outer, metabolically most active, cell layers. Adjacent untargeted cells would have continued to proliferate since they experienced no crossfire from their targeted neighbours. Consequently, growth would be unaffected. [$^{211}$At]MABG has already been shown to be significantly more toxic than [$^{131}$I]MIBG to neuroblastoma cell monolayers (Strickland et al, 1994). The results presented here confirm this effect in small spheroids. Its efficacy in large spheroids was not determined.

For the long-range β-emitting conjugate ([$^{131}$I]MIBG), the size dependence of toxicity was opposite to that of [$^{123}$I]MIBG and [$^{125}$I]MIBG, i.e. small spheroids were less vulnerable to [$^{131}$I]MIBG than large ones. This may be due to the dissipation of more β-decay energy outside small target volumes, as predicted from microdosimetric considerations (Wheldon et al, 1991; O'Donoghue et al, 1995) and in agreement with previous experimental findings (Gaze et al, 1992). This explanation is supported

**Figure 6** Growth curves of 400 μm-diameter SK-N-BE(2c) spheroids after treatment with (A) [$^{123}$I]MIBG, (B) [$^{125}$I]MIBG and (C) [$^{131}$I]MIBG. ●, Control; ○, 0.1 MBq ml$^{-1}$; □ 1 MBq ml$^{-1}$; ■, 2 MBq ml$^{-1}$; ▲, 3 MBq ml$^{-1}$; ◆, 4 MBq ml$^{-1}$. The ordinate is the common logarithm of the spheroid volume in units of (μm)$^3$. Each point represents the median of 24 measurements by the results of our data obtained from monolayer studies which indicated that, of the radioiodine isotopes examined, $^{131}$I was the least effective inhibitor of colony formation. Because of the planar geometry of cellular monolayers, most of the decay energy would have been deposited above and below the plane of the cells.

These findings are in agreement with the results of a recent study by Weber et al (1996), who compared the therapeutic effectiveness of $^{131}$I and $^{125}$I-labelled MIBG in neuroblastoma spheroids. Using a mathematical model, the radiation dose rates within small spherical tumours could be calculated. These calculations predict that higher mean dose rates can be achieved by [$^{125}$I]MIBG than [$^{131}$I]MIBG in tumour diameters up to 100 μm. However, in larger tumours, the mean dose rates achieved by $^{131}$I-labelled MIBG

Table 1 Relative toxicity of alternative radionuclides conjugated to benzylguanidine

| Radiolabel | Radiotoxicity[a] | | |
|---|---|---|---|
| | Monolayer | Small spheroid | Large spheroid |
| $^{131}I$ | + | + | ++ |
| $^{123}I/^{125}I$ | ++ | ++ | 0 |
| $^{211}At$ | +++[b] | +++ | ND |

[a]Number of + signs allow comparison of rank order of inhibitory potency within columns or rows. They do not indicate proportional effectiveness. 0 indicates absence of growth inhibition at the concentrations of radioactivity used in this study. [b]Toxicity of $^{211}At$ to cells grown in monolayer taken from Strickland et al, 1994. ND, not determined.

Table 2 Comparison of the toxicities of radionuclide conjugates of MIBG in terms of administered radioactivity in kBq $ml^{-1}$

| Endpoint | Activity (kBq $ml^{-1}$) required to achieve end point | | | |
|---|---|---|---|---|
| | $^{211}At$ | $^{123}I$ | $^{125}I$ | $^{131}I$ |
| Clonogenic survival[a] | 0.22 | 480 | 300 | – |
| Spheroid regression[b] | 1.37 | 1414 | 320 | 3460 |

[a]Survival fraction of 0.1. [b]No spheroid regrowth by 20 days.

Table 3 Comparison of the toxicities of radionuclide conjugates of MIBG in terms of number of atoms $ml^{-1}$

| Endpoint | No of atoms $\times 10^{10}$ required to achieve end point | | | |
|---|---|---|---|---|
| | $^{211}At$ | $^{123}I$ | $^{125}I$ | $^{131}I$ |
| Clonogenic survival[a] | 0.0009 | 3.50 | 230 | – |
| Spheroid regression[b] | 0.00513 | 9.68 | 241 | 346 |

[a]Survival fraction of 0.1. [b]No spheroid regrowth by 20 days.

would be up to eightfold higher. These predictions were borne out by in vitro experiments, which found [$^{125}I$]MIBG to be superior to [$^{131}I$]MIBG in treating small spheroids but that, as spheroid size increased, this advantage was lost and [$^{131}I$]MIBG became the more effective radiopharmaceutical.

To allow a quantitative comparison of the relative effectiveness of each isotope, the dose in kBq $ml^{-1}$ and in atoms $ml^{-1}$ was calculated for each analogue of MIBG. As Table 2 indicates, on a kBq $ml^{-1}$ basis, [$^{211}At$]MABG was substantially more toxic than any of the radioiodinated species. Of the iodinated MIBG molecules, the rank order of toxicity was $^{125}I > {}^{123}I > {}^{131}I$. On conversion of these activities to the number of atoms present per ml, $^{211}At$ remains the most toxic; however, $^{123}I$ rather than $^{125}I$ was the most effective iodine species (Table 3). These calculations highlight the potential of $^{123}I$ over $^{125}I$ for targeted radiotherapy. Although it is less efficient in the production of DNA double-strand breaks [0.74 compared with 1.00 per cell per decay for $^{125}I$ (Makrigiorgos et al, 1992)], the number of atoms of $^{123}I$ present in a given activity is approximately 100-fold fewer. Therefore, significantly lower molar amounts of [$^{123}I$]MIBG would be required to deliver a dose of targeted radiation. It is predicted that the administration of low concentrations of MIBG should decrease passive, relative to active, drug uptake, thereby enhancing the therapeutic ratio (Mairs et al, 1995). Furthermore, the number of radioactive atoms administered should be as low as possible to minimize the dose to normal organs that are capable of uptake with long-term retention (e.g. thyroid).

The results of this in vitro study suggest that short-range conjugates of MIBG could be effective in the treatment of metastatic neuroblastoma. A prerequisite to examining their potential in vivo is the availability of a realistic murine model of micrometastatic neuroblastoma. Recent studies using severe combined immune deficient (SCID) mice have demonstrated dissemination of human neuroblastoma cells to the liver, adrenal glands and bone marrow. Metastases in different organs had characteristic histopathology, and those in the bone marrow presented as syncytia-like cell aggregates – features typically seen in patients (Bogenmann, 1996). Such a model could be used to investigate the efficacy of $^{123}I$-, $^{125}I$- and $^{211}At$-labelled benzylguanidine conjugates in vivo.

The observation that Auger electron emitters conjugated to benzylguanidine are capable of killing SK-N-BE(2c) neuroblastoma cells raises important questions about the subcellular location of MIBG and the mechanism of cell death. Several hypotheses could explain these results. MIBG may localize in the nucleus of neuroblastoma cells. It is also possible that there are other subcellular targets for Auger electrons, such as the mitochondria. Alternatively, particles other than Auger electrons emitted during the decay of $^{123}I$ and $^{125}I$ may have sufficient range to penetrate into the nucleus of a targeted cell. A fourth possibility is that MIBG labelled with Auger electron emitters mediates cell kill through apoptosis triggered by a novel mechanism.

The conventional opinion is that the critical cellular target for ionizing radiation damage is nuclear DNA. Therefore, ultra-short-range radionuclides, such as Auger electron emitters, should be toxic only if delivered to the nucleus of the target cell (Charlton, 1986). This has been confirmed by in vitro studies – using extracellular $Na^{125}I$, cytoplasmic [$^{125}I$]iododihydrorhodamine and nuclear $^{125}$IUdR – which demonstrated that significant toxicity was associated only with the nuclear located $^{125}I$ (Kassis et al, 1987). Similar results have also been observed in vivo by Link and colleagues, who compared the therapeutic efficacy of $^{125}I$ and $^{211}At$-labelled methylene blue to treat mice bearing malignant melanoma. Only $^{211}At$-labelled methylene blue produced significant therapeutic effects. As the subcellular fate of this targeting agent is cytoplasmic, it was concluded that $^{125}I$-labelled methylene blue was too far away from the genome to produce cytotoxicity (Link et al, 1989). Although subcellular localization studies have demonstrated that MIBG concentrates mainly in the cytoplasm of neuroblastoma cells (Gaze et al, 1991; Clerc et al, 1993), the fixation procedures employed in these studies may have caused a redistribution of the drug (Smets et al, 1991). The demonstration that significant cell kill can be achieved with $^{125}I$ or $^{123}I$-labelled MIBG could represent evidence for a nuclear localization of MIBG. The amount of drug accumulated at this site may be undetectable by conventional means but nevertheless capable of delivering a toxic dose of radiation to the cell nucleus.

Alternatively, MIBG conjugated to Auger electron emitters could be cytotoxic through damage to the mitochondria. Subcellular localization studies have identified mitochondria as sites of MIBG accumulation (Gaze et al, 1991). Mitochondrial toxicity could result from disruption of inner mitochondrial membrane proteins or by direct effects on the mitochondrial genome. Indeed,

 © Cancer Research Campaign 1998

mitochondrial DNA is susceptible to radiation damage because of its limited capacity for repair (Tritschler and Medori, 1993).

It is also conceivable that some particles emitted during the decay of $^{123}$I and $^{125}$I may have sufficient range to reach genomic DNA of a targeted cell despite cytoplasmic or perinuclear localization. Although the entire Auger and Coster–Kronig electron spectra for these radionuclides have not been measured experimentally, calculations using theoretical transition rates and energies indicate that both isotopes emit some electrons with ranges of the order of the radius of a mammalian cell (Howell, 1992; Sastry, 1992). Assuming a cytoplasmic location for MIBG, it is possible that such emissions would deliver significant doses of radiation to the nucleus. However, this possibility is not supported by the classical experiments of Kassis and colleagues described earlier (Kassis et al, 1987).

A more exotic possibility is that Auger electron irradiation of cytoplasmic targets achieves cell kill by an indirect route, such as the triggering of apoptosis in susceptible cells. Some workers have reported that apoptosis-mediated cell death may result from membrane damage and the consequent generation of ceramide (Jarvis et al, 1994; Obeid et al, 1994) and that this process may be initiated by ionizing radiation damage to cell membranes (Haimovitz-friedman, 1994). We intend to investigate this possibility using DNA-targeted or cell-membrane-targeted Auger emitters ($^{125}$I incorporated in IUDR or conjugated to concanavilin A) to treat neuroblastoma cells which differ in their capacity for apoptosis and expect to be able to determine the role of membrane irradiation in radiation cell killing in apoptosis competent cells. These experiments should provide a definitive evaluation of the role of apoptosis and of membrane irradiation in cell kill by Auger electron-emitters.

Whatever the detailed mechanisms, it is clear that these alternative isotopes have potential in the treatment of neuroblastoma. Short-range Auger electron emitters are suited to the treatment of single cells or small clumps and longer range β-emitters to larger cell masses. Alpha-emitters are of very high molecular potency in treating single cells or small neuroblastoma spheroids, though their efficacy in the treatment of larger cellular aggregates is not yet evaluated. These results suggest that 'combination cocktails' of radionuclides may be beneficial in the treatment of disseminated cancer where a targeting agent exists for which alternative radioconjugates can be made. Benzylguanidine targeting of radiohalogens to neuroblastoma cells may be the first example of a strategy with wide application in targeted radiotherapy.

### CONCLUSION

The experiments reported demonstrate the theoretically expected relationships between radiotoxicity, radionuclide emission characteristics and the geometrical configuration of the target cell kill population, while posing some questions about mechanisms of cell kill by Auger electron emitters. These results suggest that the combined use of short-range and long-range particle-emitting radionuclide conjugates of benzylguanidine could enhance the therapeutic efficacy of targeted radiotherapy in neuroblastoma patients with disseminated disease.

### ACKNOWLEDGEMENTS

This study was supported by generous grants from the Cancer Research Campaign and the Neuroblastoma Society.

### REFERENCES

Beidler JL, Roffler-Tarlov S, Schachner M and Freedman LS (1978) Multiple neurotransmitter synthesis by human neuroblastoma cell lines and clones. *Cancer Res* **38**: 3751–3757

Bogenmann E (1996) A metastatic neuroblastoma model in SCID mice. *Int J Cancer* **67**: 379–385

Bruchelt G, Girgert R, Buck J, Wolburg H, Niethammer D and Treuner J (1988) Cytotoxic effects of *m*-[$^{131}$I]- and *m*-[$^{125}$I]iodobenzylguanidine on the human neuroblastoma cell lines SK-N-SH and SK-N-LO. *Cancer Res* **48**: 2993–2997

Charlton DE (1986) The range of high LET effects from $^{125}$I decays. *Radiat Res* **107**: 163–171

Clerc J, Halpern S, Fourre C, Omri F, Briancon J, Eusset J and Fragu P (1993) SIMS microscopy imaging of the intratumour biodistribution of metaiodobenzylguanidine in the human SK-N-SH neuroblastoma cell line xenografted into nude mice. *J Nucl Med* **34**: 1565–1570

DeKraker J, Hoefnagel CA, Caron H, Valdes Olmos RA, Zsiros J, Heij HA and Voute PA (1995) First line targeted radiotherapy, a new concept in the treatment of advanced stage neuroblastoma. *Eur J Cancer* **31A**: 600–602

Gaze MN, Huxham IM, Mairs RJ and Barrett A (1991) Intracellular localization of metaiodobenzylguanidine in human neuroblastoma cells by electron spectroscopic imaging. *Int J Cancer* **47**: 875–880

Gaze MN, Mairs RJ, Boyack SM, Wheldon TE and Barrett A (1992) $^{131}$I-metaiodobenzylguanidine therapy in neuroblastoma spheroids of different sizes. *Br J Cancer* **66**: 1408–1052

Gaze MN, Wheldon TE, O'Donoghue JA, McNee SE, Simpson E and Barrett A (1995) Multi-modality megatherapy with [$^{131}$I]metaiodobenzylguanidine, high dose melphalan and total body irradiation with bone marrow rescue: feasibility study of a new strategy for advanced neuroblastoma. *Eur J Cancer* **31A**: 252–256

Gaze MN and Wheldon TE (1996) Radiolabelled MIBG in the treatment of neuroblastoma. *Eur J Cancer* **32A**: 93–96

Gelfand MJ (1993) Meta-iodobenzylguanidine in children. *Semin Nucl Med* **23**: 231–242

Guerreau D, Thedrez P, Fritsch P, Saccavini J-C, Metivier H, Nolibe D, Masse R, Coornaert S and Chatal J-F (1990) *In vitro* therapeutic targeting of neuroblastomas using $^{125}$I-labelled meta-iodobenzylguanidine. *Int J Cancer* **45**: 1164–1168

Haimovitz-friedman A, Kan C-C, Ehleiter D, Persaud RS, McLoughlin M, Fuks Z and Kolesnick RN (1994) Ionizing radiation acts on cellular membranes to generate ceramide and initiate apoptosis. *J Exp Med* **180**: 525–535

Hartmann O, Lumbroso J, Lemerle J, Schulmberger M, Ricard M, Aubert B, Coonaert S, Olive D, Delumley L and Parmentier C (1987) Therapeutic use of $^{131}$I-metaiodobenzylguanidine (MIBG) in neuroblastoma: a phase II study in nine patients. *Med Pediatr Oncol* **15**: 205–211

Hoefnagel CA, Smets L, Voute PA and De Kraker J (1991) Iodine-125-MIBG therapy for neuroblastomas. *J Nucl Med*. **32**: 361–362

Hoefnagel CA (1994) Metaiodobenzylguanidine and somatostatin in oncology: role in the management of neural creast tumours. *Eur J Nucl Med* **21**: 561–581

Howell RW (1992) Radiation spectra for Auger electron emitting radionuclides. Report No 2 of AAPM-Nuclear-Medicine-Task-Group No 6. *Med Phys* **19**: 1371–1383

Humm JL (1986) Dosimetric aspects of radiolabelled antibodies for tumour therapy. *J Nucl Med* **27**: 1490–1497

Jarvis WD, Kolesnick RN, Fornari FA, Traylor RS, Gewirtz DA and Grant S (1994) Induction of apoptotic DNA damage and cell death by activation of the sphingomyelin pathway. *Proc Natl Acad Sci USA* **91**: 73–77

Kassis AI, Fayad F, Kinsey BM, Sastry KSR, Taube RA and Adelstein SJ (1987) Radiotoxicity of $^{125}$I in mammalian cells. *Radiat Res* **111**: 305–318

Larsen RH, Bruland OS, Hoff P, Alstad J, Lindmo T and Rofstad EK (1994) Inactivation of human osteosarcoma cells *in vitro* by 211-At-TP-3 monoclonal antibody: comparison with astatine-211-labelled bovine serum albumin, free astatine-211 and external-beam X rays. *Radiat Res* **139 (2)**: 178–184

Link EM, Brown I, Carpenter RN and Mitchell JS (1989) Uptake and therapeutic effectiveness of $^{125}$I- and $^{211}$At-methylene blue for pigmented melanoma in an animal model system. *Cancer Res* **49**: 4332–4337

Link EM and Carpenter RN (1990) $^{211}$At-methylene blue for targeted radiotherapy of human melanoma xenografts: treatment of micrometastases. *Cancer Res* **50**: 2963–2967

Link EM and Carpenter RN (1992) $^{211}$At-Methylene blue for targeted radiotherapy of human melanoma xenografts: treatment of cutaneous tumours and lymph node metastases. *Cancer Res* **52**: 4385–4390

Link EM, Carpenter RN and Hansen G (1996) [$^{211}$At]Methylene blue for the targeted radiotherapy of human melanoma xenografts: dose fractionation in the treatment of cutaneous tumours. *Eur J Cancer* **32A**: 1240–1247

Mairs RJ, Angerson W, Gaze MN, Murray T, Babich JW, Reid R and McSharry C (1991) The distribution of alternative agents for targeted radiotherapy within neuroblastoma spheroids. *Br J Cancer* **63**: 404–409

Mairs RJ, Gaze MN, Watson DG, Skellern GG, Constable P, McKellar K, Owens J, Vaidyanathan G and Zalutsky MR (1994*a*) Carrier-free $^{131}$I-meta-iodobenzylguanidine: comparison of production from meta-diazobenzylguanidine and from meta-trimethylsilylbenzylguanidine. *Nucl Med Commun* **15**: 268–274

Mairs RJ, Livingstone A, Gaze MN, Wheldon TE and Barrett A (1994*b*) Prediction of accumulation of $^{131}$I-meta-iodobenzylguanidine in neuroblastoma cell lines by means of reverse trancription and poymerase chain reaction. *Br J Cancer* **70**: 97–101

Mairs RJ, Cunningham SH, Russell J, Armour A, Owens J, McKellor K and Gaze MN (1995) No-carrier-added iodine-131-MIBG: evaluation of a therapeutic preparation. *J Nucl Med* **36**: 1088–1095

Makrigiorgos GM, Berman RM, Baranowska-kortylewicz J, Bump E, Humm JL, Adelstein SJ and Kassis AI (1992) DNA damage produced in V79 cells by DNA-incorporated Iodine-123: a comparison with iodine-125. *Radiat Res* **129**: 309–314

Mastrangelo R (1987) Editorial: the treatment of neuroblastoma with $^{131}$I-MIBG. *Med Pediatr Oncol* **15**: 157–158

Mastrangelo R, Tornesello A, Riccardi R, Lasorella A, Mastrangelo S, Mancini A, Rufini V and Troncone L (1995) A new approach in the treatment of stage IV neuroblastoma using a combination of [$^{131}$I]metaiodobenzylguanidine (MIBG) and cisplatin. *Eur J Cancer* **31A**: 606–611

Obeid LM, Linardic CM, Karolak LA and Hannun YA (1994) Programmed cell death induced by ceramide. *Science* **259**: 1769–1771

O'Donoghue JA, Bardies M and Wheldon TE (1995) Relationships between tumour size and curability for uniformly targeted therapy with beta-emitting radionuclides. *J Nucl Med* **36**: 1902–1909

Rutgers M, Buitenhuis C, Hoefnagel CA, Voute PA and Smets LA (1994) Therapeutic efficacy of $^{125}$I and $^{131}$I-MIBG in macroscopic and microscopic tumours of neural-crest origin (abstract). In *Proceedings of the 2nd European Symposium on Neuroblastoma: Recent Advances in Clinical, Cellular and Genetic Analysis*, 9–11 June, Heidelberg.

Sastry KSR (1992) Biological effects of the Auger emitter I-125 – a review. Report No 1 of AAPM-Nuclear-Medicine-Task-Group No 6. *Med Phys* **19**: 1361–1370

Schwabe D, Sahm S, Gerein V, Happ J, Kropp-v-Rabenau H, Maul F, Baum RP, Mangegold K, Nitz C, Hor G and Kornhuber B (1987) $^{131}$I-metaiodobenzylguanidine therapy of neuroblastoma in childhood. One year of therapeutic experience. *Eur J Paediatr* **146**: 246–250

Senekowitsch R, Weber W, Meindl J, Bruchelt G, Kretschko J and Pabst HW (1992) Experimental studies for therapy of neuroblastoma micrometastases with radioiodinated MIBG using multicellular tumour spheroids (Abstract No 144). *J Nucl Med* **33**: 859

Shapiro B and Gross MD (1987) Radiochemistry, biochemistry, and kinetics of $^{131}$I-metaiodobenzylguanidine (MIBG) and $^{123}$I-MIBG: clinical implications of the use of $^{123}$I-MIBG. *Med Pediatr Oncol* **15**: 170–177

Sisson JC, Hutchinson RJ and Shapiro B (1989) Markers and goals for $^{131}$I-MIBG treatment of neuroblastoma. In *Biology of Radionuclide Therapy*, DeNardo GL, Lewis JP, Raventos A and Burt RW (eds), pp. 85–93. American College of Nuclear Physicians, Publications Department: Washington, DC

Sisson JC, Hutchinson RJ, Shapiro B, Zasadny KR, Normolle D, Weiland DM, Wahl RL, Singer DA, Mallette SA and Mudgett EE (1990) Iodine-125-MIBG to treat neuroblastoma: preliminary report. *J Nucl Med* **31**: 1479–1485

Sisson JC, Shapiro B, Hutchinson RJ, Zasadny KR, Mallette S, Mudgette EE and Weiland DM (1991) Treatment of neuroblastoma with [$^{125}$I]Metaiodobenzylguanidine. *J Nucl Biol Med* **35**: 255–259

Sisson JC, Shapiro B, Hutchinson RJ, Shulkin BL and Zempel S (1996) Survival of patients with neuroblastoma treated with $^{125}$I-MIBG. *Am J Clin Oncol Cancer Clin Trials* **19**: 144–148

Smets LA, Janssen M, Rutgers M, Ritzen K and Buitenhuis C (1991) Pharmacokinetics and intracellular distribution of the tumour targeted radiopharmaceutical *m*-iodo-benzylguanidine in SK-N-SH neuroblastoma and PC-12 phaeochromocytoma cells. *Int J Cancer* **48**: 609–615

Soule HD, Vazquez J, Long A, Albert S and Brennan M (1973) A human cell line from a pleural effusion derived from a breast carcinoma. *J Natl Can Inst* **51**: 1409–1416

Strickland DK, Vaidyanathan G and Zalustsky MR (1994) Cytotoxicity of α-particle-emitting *m*-[$^{211}$At] astatobenzylguanidine on human neuroblastoma cells. *Cancer Res* **54**: 5414–5419

Tritschler H-J and Medori R (1993) Mitochondrial DNA alterations as a source of human disorders. *Neurology* **43**: 280–288

Vaidyanathan G and Zalutsky MR (1992) 1-(*m*-[$^{211}$AT]astatobenzyl)-guanidine: synthesis via astato demetalation and preliminary *in vitro* and *in vivo* evaluation. *Bioconjugate Chem* **3**: 499–503

Vaidyanathan G and Zalutsky MR (1993) No-carrier-added synthesis of *meta*-[$^{131}$I]iodobenzylguanidine. *Appl Radiat Isot* **44**: 621–628

Vergote I, Larsen RH, De Vos L, Nesland JM, Bruland O, Bjorgum J, Alstad J, Trope C and Nustad K (1992) Therapeutic efficacy of the alpha-emitter $^{211}$At bound on microspheres compared with $^{90}$Y and $^{32}$P colloids in a murine intraperitoneal tumour model. *Gynecol Oncol* **47**: 366–372

Voute PA, Hoefnagel CA and De Kraker J (1988) $^{131}$I metaiodobenzylguanidine in diagnosis and treatment of neuroblastoma. *Bull Cancer* **75**: 107–111

Weber W, Weber J and Senekowitsch-Schmidtke S (1996) Therapeutic effect of *m*-[$^{131}$I]- and *m*-[$^{125}$I] iodobenzylguanidine on neuroblastoma multicellular tumour spheroids of different sizes. *Cancer Res* **56**: 5428–5434

Weiland D, Wu J, Brown L, Manger TE, Swanson DP and Bierwaltes WH (1980) Radiolabelled adrenergic neurone-blocking agents: adrenomedullary imaging with [$^{131}$I]iodobenzylguanidine. *J Nucl Med* **21**: 349–353

Wheldon TE, Livingstone A, Wilson L, O'Donoghue J and Gregor A (1985) The radiosensitivity of human neuro-blastoma cells estimated from regrowth curves of multicellular tumor spheroids. *Br J Radiol* **58**: 661–664

Wheldon TE, O'Donoghue JA, Barrett A and Michalowski AS (1991) The curability of tumours of differing sizes by targeted radiotherapy using I-131 and Y-90. *Radiother Oncol* **21(2)**: 91–99

© Cancer Research Campaign 1998